# Comparative in vitro study of the accuracy of impression techniques for dental implants: Direct technique with an elastomeric impression material versus intraoral scanner

**DOI:** 10.4317/medoral.22822

**Published:** 2018-12-24

**Authors:** Cristina Rech-Ortega, Lucía Fernández-Estevan, Mª Fernanda Solá-Ruíz, Rubén Agustín-Panadero, Carlos Labaig-Rueda

**Affiliations:** 1 DDS, PhD. Associate Professor, Department of Dental Medicine, Faculty of Medicine and Dentistry, University of Valencia, Valencia, Spain; 2 DDS, PhD, MD. Adjunct Professor, Department of Dental Medicine, Faculty of Medicine and Dentistry, University of Valencia, Valencia, Spain; 3 DDS, PhD. Adjunct Professor, Department of Dental Medicine, Faculty of Medicine and Dentistry, University of Valencia, Valencia, Spain; 4 DDS, PhD, MD. Professor, Department of Dental Medicine, Faculty of Medicine and Dentistry, University of Valencia, Valencia, Spain

## Abstract

**Background:**

The aim of this study was to compare a conventional technique (elastomeric impression material - EIM) and a digital technique (scanner digital model – SDM) on a six-analog master model (MM) to determine which was the most exact.

**Material and Methods:**

Twenty impressions were taken of a master model (EIM) and twenty scanned impressions (SDM) (True Definition). A coordinate measuring machine (CMM) was used to measure the distances between adjacent analogues (1-2, 2-3, 3-4, 4-5, 5-6), intermittently positioned analogues (1-4, 3-6) and the most distal (1-6). Reference values were established from the master model, which were compared with the two impression techniques. The significance level was established as 5% (*p*<0.05).

**Results:**

The precision of each technique was compared with MM. For adjacent analogues (1-2), no significant differences were found between EIM-MM (*p*=0,146). For intermittently positioned analogues (1-4), SDM did not show significant differences with MM (*p*=0.255). For the distance between distal analogues (1-6), significant differences were found between both techniques and MM (*p*=0.001).

**Conclusions:**

In a clinical situation with < three implants, EIM is more exact than SDM, but in cases of four implants SDM is more exact. For rehabilitations (> four implants), neither technique can be considered accurate although error falls within the tolerance limits established in the literature (30-150µm).

** Key words:**Digital workflow, full arch scan, intraoral scanner, CAD/CAM, polyether impression, accuracy.

## Introduction

The design and fabrication of implant-supported prosthetic structures requires the faithful reproduction of anatomical details and the correct positioning of the implants in the jaw. To do this, it is essential to take impressions using silicones or polyethers that will provide a working model that is as precise and exact as possible and will lead to a prosthetic structure with adequate passive fit (1,2).

Until recently, implant-supported fixed prostheses were fabricated using an impression-taking and casting system but this often failed to provide correct passive fit due to the phenomena of contraction and expansion produced during the casting process. This can cause loosening or fracture of the fixing screw, or trigger a loss of implant osseointegration (3).

In recent years, the introduction of digital workflow has brought about a revolution in the field of dentistry, particularly in relation to the fabrication of prosthetic structures, whether tooth- or implant-supported (4). Digital techniques have allowed greater management of technical errors in metal-casting, making it easier to achieve a correct prosthetic fit.

This evolution has been accompanied by the development of digital cameras and intraoral scanners (5-9), which register data directly from the oral cavity. These systems represent an improvement in patient comfort as they avoid the need to place impression-taking materials in the mouth. They also avoid the errors of dimensional stability suffered by elastomeric materials (10-13).

However, the new digital techniques have come up against some problems when it comes to impression-taking for implant-supported prostheses. For one-piece prostheses, the outcomes are adequate, but not for complete arch rehabilitations (14). For this reason, research and development of new intraoral scanners has sought to improve on the precision of conventional impression procedures using elastomeric materials.

This study aimed to compare two impression techniques for analogue implants: the direct (or pick-up) technique with the elastomeric material polyether, and a digital scanner (True Definition, 3M ESPE), in order to determine which is the more accurate technique.

## Material and Methods

Master model fabrication: A cylindrical model was fabricated from titanium supporting six analogue implants with internal hex connections of 4.1 mm diameter (Certain Biomet 3i), numbered from 1 to 6 from left to right.

Impression-taking techniques: Impressions were taken of the master model using two different techniques, conventional direct technique using polyether (elastomeric impression material – EIM), better known as pick-up impression, and a digital impression technique using an intraoral scanner (True Definition, 3M ESPE) (scanner digital model – SDM).

For the first group (EIM), twenty individual impression trays were made from photopolymerizable acrylic resin (Revorlight, Techim Group) with six perforations corresponding to the implant analogues. Impression copings were screwed onto the master model analogues and polyether (Impregum PentaSoft, 3M ESPE) was injected into the tray around them. When set, the tray was removed and replicas were cast in Type IV plaster (Vel-MixStone, Kerr). This process was repeated 20 times to produce 20 working models.

For the digital technique (SDM), six scan bodies (Internal Certain, 4.1mm diameter, Core3d centres) were screwed to the master model analogues, applying a layer of silicone-based permanently soft relining material (Ufi Gel SC, VOCO), and over this a fine layer of titanium dioxide (3M High-Resolution Scanning Spray) to avoid any reflection during the scanning process.

**Figure 1:**
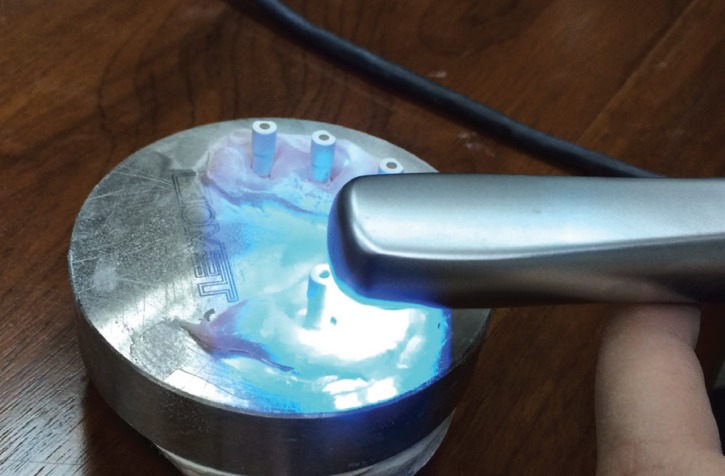
Scanning the master model with the intraoral camera (True Definition, 3M ESPE).

When ready, the model was scanned (Fig. 1), obtaining a digital model displayed on the scanner monitor. The data obtained, known as STL files (Standard Tessellation Language, a standardized file format native to 3D system software that describes the surface geometry of a three-dimensional object) were sent online to the laboratory. The process was repeated 20 times to obtain 20 digital models.

In this way, the entire sample consisted of the master model (control group), 20 physical models obtained with the direct technique (EIM) and 20 digital models (SDM) obtained using the True Definition scanner (3M ESPE).

**Figure 2:**
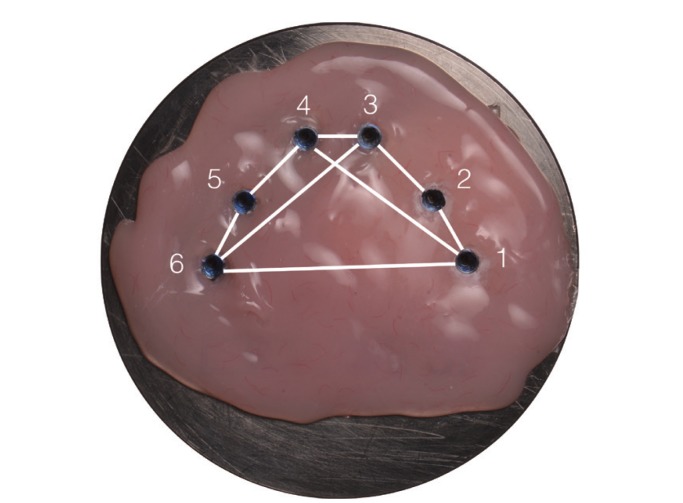
Measurement of distances between master model analogues. Positions of analogues on master model, numbered from 1 to 6. In blue: measuring sequence from right to left. In red: the distances measured between adjacent analogues (1-2, 2-3, 3-4, 4-5, 5-6), intermittently positioned analogues (1-4, 3-6) and the most distally positioned analogues (1-6).

**Figure 3:**
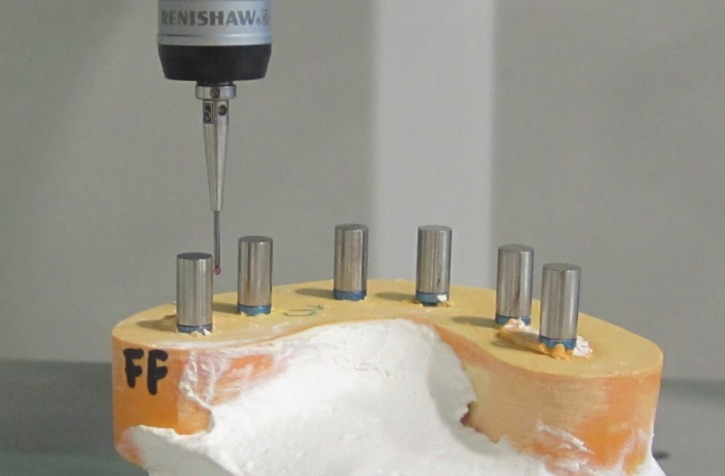
Measurement of distances (EIM). Plaster model produced using EIM impression technique with scan bodies screwed in place, set in coordinate measurement machine (CMM).

**Figure 4:**
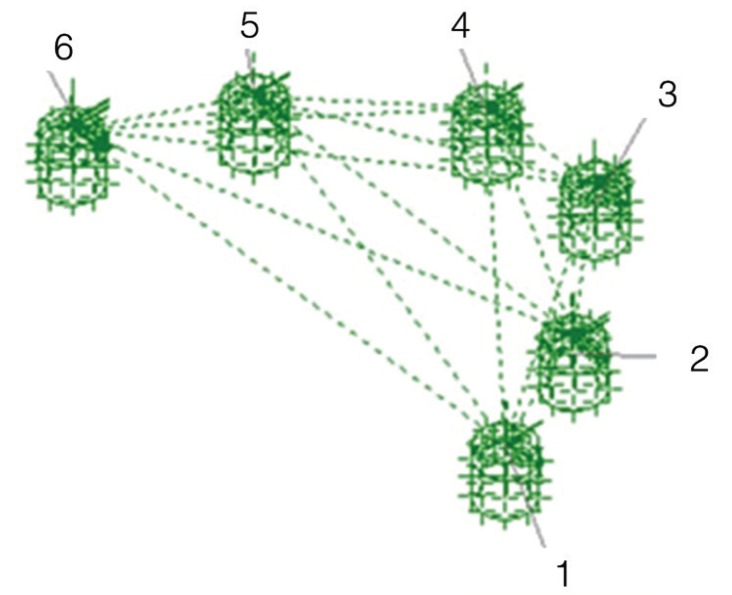
Measurement of distances (SDM). Images of scan bodies captured using the True Definition® scanner (3M® ESPE: USA) showing distances measured between analogues.

Measuring system: To determine which of the techniques was the most accurate, reference data composed of three axes XYZ were taken from the master model by means of a coordinate measuring machine. Six cylindrical scan bodies (Biomet 3i) were screwed onto the analogues and the following distances were measured: between adjacent analogues from center to center: 1-2, 2-3, 3-4, 4-5, 5-6; between intermittently positioned analogues: 1-4, 3-6; between the most distal analogues: 1-6 (Fig. 2). This process was repeated for the 20 physical models (Fig. 3). When the laboratory had received the digital models of the scan bodies (Laboratorio Garbident), CAD software including an implant library was used (Dental Designer, 3Shape) to take measurements between the analogues (Fig. 4).

Five parameters (X, Y, Z, module XY, module XYZ) were calculated for each distance 1-2, 2-3, 3-4, 4-5, 5-6, 1-4, 3-6, 1-6 measured from the 20 physical models (EIM) and the master model. For the twenty digital models (SDM), software provided XYZ distance modules directly. So for each EIM physical model and the master model, 40 XYZ module distance measurements were registered: five parameters for eight distances, and eight measurements for each digital model. In addition, for the master model and the EIM physical models, three repetitions of each distance per model were made, making a total of 2.680 measurements, as follows.

- Master model: 1 model x 8 distances x 5 parameters x 3 repetitions = 120 measurements.

- EIM: 20 models x 8 distances x 5 parameters x 3 repetitions = 2,400 measurements.

- SDM: 20 models x 8 distances x 1 parameter x 1 repetition = 160 measurements.

Statistical analysis: Statistical analysis applied Student’s t-test and analysis of variance (ANOVA). The significance level was 5% (*p*<0.05).

## Results

Results are expressed in terms of the accuracy of the two impression techniques used – EIM (direct technique) and SDM (True Definition intraoral scanner, 3M ESPE) – in comparison with the master model (control group) in order to determine which technique obtained values closest to the master model. The XYZ module parameter was analyzed as this indicated the real distances in millimeters between the analogue centers.

For each distance between analogues, the mean measurements obtained were calculated for the master model, EIM and SDM ( Table 1). Then differences between master model mean distances and each impression technique were calculated applying Student’s t-test to obtain a p-value used to determine whether the XYZ module for each distance could be accepted as equal to the master model or not ( Table 2).

**Table 1:**
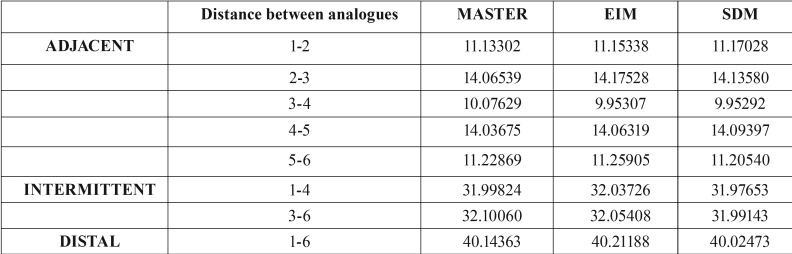
Mean measurements taken with the two techniques (EIM, SDM) and reference measurements of master model (control): XYZ module parameter, for each distance between adjacent analogues (1-2, 2-3, 3-4, 4-5, 5-6), intermittently positioned analogues (1-4, 3-6), and distal analogues (1-6).

**Table 2:**
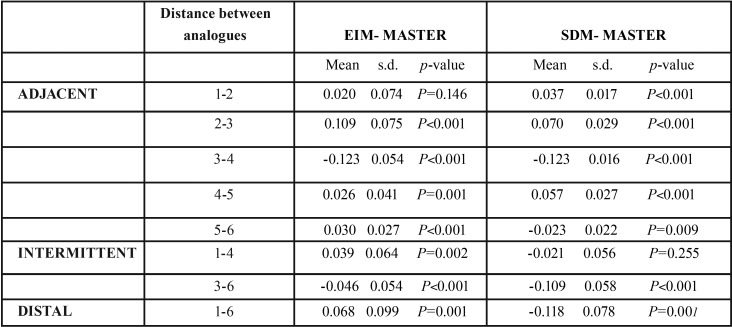
Table shows differences between master model mean measurements and measurements obtained with each impression technique (EIM and SDM), standard deviation and p-value, for the XYZ module parameter for each distance between adjacent analogues (1-2, 2-3, 3-4, 4-5, 5-6), intermittently positioned analogues (1-4, 3-6), and the most distally positioned analogues (1-6). Statistical significance level was 5% (*p*<0.05). *P*-values >0.05 are highlighted in grey, indicating no significant difference between the impression technique and the master model.

As shown in  Table 2, no statistically significant differences were found for distances between adjacent analogues between EIM data and the master model for the 1-2 distance, (*p*=0.146; mean= 0.0203; standard deviation (s.d.) = 0.074). Between intermittently positioned analogues, SDM did not present significant differences, obtaining values close to the master model for the distance 1-4 (*p*-value = 0.255; mean = -0.021; s.d. = 0.056).

Statistically significant differences were found between results obtained by both techniques in comparison with the master model for the distance between distal analogues (1-6) and all other distances (2-3,3-4,4-5,5-6,3-6) (*p*<0.05).

## Discussion

Measurement of distances between master model analogues (control group) and distances on physical models was performed using a coordinate measurement machine (CMM) with a measuring accuracy within 4.2μm. Various researchers have used this system, with accuracy ranges of between 1 and 5μm (9,12,15-17). Other measurement methods include: profile projectors with accuracy within 2μm (18,19); optical microscopes with accuracy within 0.5 and 2μm (20,21); and table-top scanners (22,23).

To validate the measurement method used (CMM), its reproducibility was analyzed on the master model (control group) obtaining coefficients of variation that varied between 0.00027 and 0.00385% – figures well below the 1% reference value – which were considered compatible with almost perfect reproducibility.

Measurements of digital models (SDM) were taken using CAD design software (Dental Designer, 3Shape) measuring the distances between analogues.

The elastomeric material used was polyether (Impregum Penta Soft, 3M ESPE), one of the most widely used impression materials in cases of prostheses supported by multiple implants, due to its rigidity and dimensional stability. Addition silicones also offer ideal characteristics as impression materials and are able to reproduce intraoral implant positions adequately (18,21).

With regard to the impression technique used, various authors (18,21-23) have affirmed that the direct technique is exact. Others, have used impression coping splinting systems to obtain greater accuracy in working models, such as rigid impression splints, or using autopolymerizable acrylic resin (for example, Duralay [Reliance Dental] or Pi-Ku-Plast [Bredent]) (20,24). However, some research affirms that there are no statistically significant differences between methods, whether splinting or not (20,25).

As for the accuracy of conventional techniques compared with digital techniques, according to the present results, conventional polyether impressions (EIM) showed a greater number of distances between adjacent analogues without significant differences from the master model (1-2,2-3,3-4,4-5,5-6) (*p*>0,05) (1/5 values for the XYZ module parameter), than the digital technique (SDM) (0/5 values). So, in a clinical case involving up to three adjacent implants, EIM can be used to obtain correct passive fit of the prosthetic structure.

Giménez (9,12,13) made several comparative studies of different intraoral scanners, iTero (Align Technologies), LavaCOS (3M ESPE), 3D Progress (Medical High Technologies), and ZFX Intrascan (Zfx GmbH), comparing them with a six-implant master model. He found that among closely positioned implants (1-2), the most exact system was iTero (Align Technologies), with mean error of 14.3μm, while for the same distance in the present study, using the scanner True Definition (3M ESPE), mean error was 37μm.

Papaspyridakos (26) compared the intraoral scanner TRIOS (3Shape) with two conventional direct impression techniques: splinted and non-splinted. He concluded that in cases of two- or three-piece bridges, the digital system was as exact as the conventional techniques, analogue splinting being preferable. But in the present study, better results were obtained for the distance 1-2 using the direct technique (EIM).

For the distances between intermittently positioned analogues (1-4), the scanner obtained better results (EIM: 0/1, SDM: 1/1 values for the parameter module XYZ) But for the 3-6 distance, neither technique was found to be exact and statistically significant differences were found in comparison with the master model (EIM and SDM: 0/1 values for the XYZ module parameter). This indicated that in a clinical situation involving four implants, the scanner can be used, but in cases with larger numbers of implants, it should be noted that the greater the distance from the reference analogue, the greater the loss of accuracy will be, and so the worse the passive fit of the finished prosthetic structure.

Giménez (9,12,13) observed that for measuring distances between intermittently positioned implants (1-4), the iTero system (Align Technologies) obtained a lower mean error than the other digital systems assayed (27.9μm), obtaining similar results to the present study (21μm).

For the distance between the two most distally positioned analogues (1-6), neither technique was found to be valid (neither EIM, nor SDM: 0/1 values for the XYZ parameter module). This indicated that in cases of full arch rehabilitation, accuracy will be lost the greater the distance from the reference analogue, in the same way as intermittently positioned analogues (3-6).

Ender (27) made a comparative study of the direct technique and two intraoral scanning systems, Cerec AC Bluecam (Sirona Dental Systems) and LavaCos (3M ESPE), concluding that there were no statistically significant differences between the conventional and the digital techniques.

For Giménez (9,12,13), in spite of the fact that iTero (Align Technologies) proved to be the most reliable digital system, he concluded, like the present study, that error increased proportionally as the distances between analogues increased.

In light of these findings, it is important to select the most suitable impression technique in each clinical case, particularly in relation to the number of implants involved, as an error at this first stage could lead to a prosthetic structure with a lack of passive fit. Given that it is not easy to achieve a good fit, a degree of tolerance has been established between implants and the structure they support. According to Bränemark this tolerance should be less than 10µm, but for other authors (28,29) the interval should be between 30 and 150µm, values that are currently considered acceptable, although the exact amount of static stress that the peri-implant bone can tolerate remains unknown. A lack of fit greater than 450µm is considered unacceptable (30,31).

In the present study, the direct technique (EIM) presented inaccuracies between adjacent implants of between 20 and 123µm, while the digital system (SDM) presented inaccuracies of 23-123µm. As for the distance between intermittently positioned implants (1-4), inaccuracy was of 39µm with EIM and 21µm with SDM, and for 3-6, 46µm with EIM and 109µm with SDM. Between the most distal implants (1-6), inaccuracy was 68µm with EIM and 118µm with SDM. So all these values fell within the range of tolerance established in the literature (30-150µm), and so can be considered acceptable.

## Conclusions

Within the limitations of the present study, it may be concluded that.

1. For adjacent analogues, the direct technique (EIM) can be considered the most accurate for the XYZ module distance 1-2 as no statistically significant differences were found (*p*=0.146) in relation to the master model. For the other distances, 2-3, 3-4, 4-5 and 5-6, neither technique was completely accurate. So, in a clinical situation involving a maximum of three implants, the direct technique is recommended.

2. Between intermittently positioned analogues 1-4, the intra oral scanner True Definition (3M ESPE) (SDM) provided accurate data, without statistically significant differences in comparison with the master model (*p*=0.255). So, in a clinical situation involving four implants, the digital technique can be considered recommendable.

3. For the 3-6 distance, both techniques obtained significantly different values from the master model; the same occurred with the two most distal analogues (distance 1-6) (*p*<0.05). So, in cases of rehabilitations involving more than four implants, neither technique can be considered accurate. However, both the techniques analyzed can be used with relative reliability, as the errors produced fell within the tolerance range established in the literature as acceptable (30-150µm), although it is advisable to make a verification splint before fabricating the definitive prosthesis.
